# Are rabid raccoons (*Procyon lotor*) ready for the rapture? Determining the geographic origin of rabies virus‐infected raccoons using RADcapture and microhaplotypes

**DOI:** 10.1111/eva.13613

**Published:** 2023-11-20

**Authors:** Matthew W. Hopken, Antoinette J. Piaggio, Zaid Abdo, Richard B. Chipman, Clara P. Mankowski, Kathleen M. Nelson, Mikaela Samsel Hilton, Christine Thurber, Mirian T. N. Tsuchiya, Jesús E. Maldonado, Amy T. Gilbert

**Affiliations:** ^1^ United States Department of Agriculture, Animal and Plant Health Inspection Service, Wildlife Services National Wildlife Research Center Fort Collins Colorado USA; ^2^ Department of Microbiology, Immunology, and Pathology Colorado State University Fort Collins Colorado USA; ^3^ United States Department of Agriculture, Animal and Plant Health Inspection Service, Wildlife Services National Rabies Management Program Concord New Hampshire USA; ^4^ Data Science Lab, Office of the Chief Information Officer Smithsonian Institution Washington DC USA; ^5^ Center for Conservation Genomics Smithsonian National Zoo and Conservation Biology Institute Washington DC USA

**Keywords:** microhaplotypes, population genetics, *Procyon lotor*, rabies virus, raccoon

## Abstract

North America is recognized for the exceptional richness of rabies virus (RV) wildlife reservoir species. Management of RV is accomplished through vaccination targeting mesocarnivore reservoir populations, such as the raccoon (*Procyon lotor*) in Eastern North America. Raccoons are a common generalist species, and populations may reach high densities in developed areas, which can result in contact with humans and pets with potential exposures to the raccoon variant of RV throughout the eastern United States. Understanding the spatial movement of RV by raccoon populations is important for monitoring and refining strategies supporting the landscape‐level control and local elimination of this lethal zoonosis. We developed a high‐throughput genotyping panel for raccoons based on hundreds of microhaplotypes to identify population structure and genetic diversity relevant to rabies management programs. Throughout the eastern United States, we identified hierarchical population genetic structure with clusters that were connected through isolation‐by‐distance. We also illustrate that this genotyping approach can be used to support real‐time management priorities by identifying the geographic origin of a rabid raccoon that was collected in an area of the United States that had been raccoon RV‐free for 8 years. The results from this study and the utility of the microhaplotype panel and genotyping method will provide managers with information on raccoon ecology that can be incorporated into future management decisions.

## 1 INTRODUCTION

Genomic tools have become a fundamental part of disease ecology research for understanding both pathogen and wildlife host movement across landscapes and tracking the source of outbreaks (Biek & Real, 2010). Many pathogen genomes have elevated evolutionary rates; thus, pathogen genomic data can be used to detect recent evolutionary events, which can inform molecular epidemiological models for source‐tracking and predict future spread (Kozakiewicz et al., 2018; Moya et al., 2004). Similarly, in host species, the characterization of highly informative genomic regions and the development of polymorphic markers help quantify population structure, gene flow, and migration patterns (Vander Wal et al., 2014). However, the application of genomic tools to identify recent movement patterns of vertebrate hosts that pose a transmission or spillover risk is more difficult due to the slower evolutionary rates, low probabilities of capturing infected hosts, and a lack of baseline genomic data for many wildlife species (Blanchong et al., 2016). Interdisciplinary efforts between geneticists, disease ecologists, and wildlife managers can take advantage of intensive wildlife disease surveillance programs to facilitate the establishment of large tissue repositories (biobanks) and apply genomic and computational pipelines for rapid and high‐throughput genotyping that can inform the epizootiology and management of wildlife diseases (Decandia et al., 2018).

High‐throughput sequencing (HTS) has revolutionized population genetics studies of non‐model organisms. Using reduced representation sequencing approaches, such as restriction site‐associated DNA sequencing (RADseq), researchers can rapidly genotype hundreds of individuals at thousands of independent genomic loci and assemble genotypes without the need for a draft genome sequence (Andrews et al., 2016). The datasets from RADseq result in thousands of single nucleotide polymorphisms (SNPs) that provide high‐resolution estimates of population structure, gene flow, and relatedness (Andrews et al., 2016; Baird et al., 2008). However, RADseq does suffer from higher per‐sample costs compared to some genotyping methods and some inherent biases due to its reliance on restriction sites (Andrews et al., 2016). Recently developed methods such as GTseq and RADcapture (Rapture) have improved the genotyping consistency and reduced the per‐sample labor and cost when utilizing management‐informative markers (Ali et al., 2015; Campbell et al., 2015).

Standard HTS genotyping methods for wildlife management and conservation, such as GTseq and Rapture, typically target bialleleic SNPs. This limited allelic diversity often requires thousands of loci per individual for enough resolution to detect population differentiation, estimate ancestry and parentage, or individual identification (Baetscher et al., 2018). To increase the per locus information content, genotyping multiple SNPs within a short HTS read can result in haplotypes that have more than two alleles, often referred to as microhaplotypes (Kidd et al., 2013, 2014; Mckinney et al., 2017). Microhaplotypes take advantage of SNPs that are in linkage disequilibrium leading to short, polymorphic DNA sequences based on each SNP that can be genotyped as multiallelic loci. Microhaplotypes were initially implemented in human forensics for individual identification (Kidd et al., 2013). However, microhaplotypes have seen increased use in ecology and conservation biology to investigate relatedness, kinship, and population structure in wildlife (Baetscher et al., 2018; Bootsma et al., 2021; Morin et al., 2021). Because fewer microhaplotypes are needed than SNPs, both GTseq and Rapture panels of a few hundred to a few thousand loci are useful for rapidly and cost‐effectively genotyping large numbers of individuals. Microhaplotypes hold potential for wildlife disease management by providing cost‐effective information about host population structure, movement, and translocations.

Rabies lyssavirus (i.e., rabies virus, RV) is a highly lethal zoonosis that can infect all mammal species (Kuzmin et al., 2012; Nel & Markotter, 2007; Troupin et al., 2016). In the United States (U.S.), the U.S. Department of Agriculture (USDA) works with federal, state, and local agencies to coordinate the management of specific wildlife RV variants to mitigate the risks posed to humans, domesticated animals, and wildlife. Since the 1990s, the USDA National Rabies Management Program (NRMP) has been funded to prevent the spread of and locally eliminate the raccoon (*Procyon lotor*) variant of rabies virus (RRV) in the eastern U.S. while also maintaining a canine rabies virus‐free status along the shared border with Mexico, accomplished through programs of oral vaccination and enhanced rabies surveillance targeting wildlife (Elmore et al., 2017; Slate et al., 2009).

Rabies virus was first reported in raccoons in Florida during the late 1940s and by the late 1970s the virus had spread to nearby states (Mclean, 1971). At that time, the unintentional human‐mediated translocation of rabid raccoons led to one of the largest epizootics of wildlife rabies in the U.S., which catalyzed support and funding for the NRMP (Rupprecht et al., 1995; Rupprecht & Smith, 1994). The NRMP conducts enhanced surveillance of RV by targeting mesocarnivores to track changes in landscape epizootiology and to evaluate the effectiveness of management toward RV control and elimination (Davis et al., 2021; Kirby et al., 2017). Long distance raccoon movements, whether natural or anthropogenic, can threaten rabies control efforts, as suggested by recent RRV incursions into eastern Canada (Nadin‐Davis et al., 2020; Trewby et al., 2017). However, managers have been lacking host population genetic tools to understand whether rabid raccoons collected in RRV‐free areas are long‐distance migrants, translocation events, or if animals are from local populations which can help estimate risk of RV spread and development of appropriate management strategies. Translocations can have detrimental outcomes on society that result in negative health and economic consequences (Chipman et al., 2008; Rosatte & Macinnes, 1989). Determining the origin of an infected individual in an area known to be free of rabies can identify early introduction and risks of onward transmission which informs management and policy toward rapid containment and possible elimination (Bird & Mazet, 2018; Martel et al., 2020).

We present an original rapid genotyping assay developed for raccoons using Rapture and polymorphic microhaplotypes. We take an in‐depth look at raccoon population structure in the northeastern U.S., an area with enzootic RRV and active rabies management. We then describe the application of this genotyping method for tracking the geographic origin of a rabid raccoon collected on Cape Cod, Massachusetts, most of which has been RRV‐free for approximately a decade. This study demonstrates how a partnership between genomic researchers and a large‐scale rabies management program to build a host species tissue archive of thousands of samples and develop genomic resources can assist with wildlife disease management.

## 2 METHODS

### 2.1 Samples

Raccoon tissue samples were collected opportunistically during routine rabies virus surveillance and management activities. From 2018 to 2021, NRMP field biologists collected ear tissue samples in silica beads from trapped, roadkill, and found‐dead specimens, which were stored at −80°C upon receipt from the field. The New York State Department of Health Wadsworth Rabies Laboratory also kindly agreed to provide brain tissue opportunistically from confirmed rabies‐negative animals collected during public health surveillance in 2021. The rabid raccoon from Cape Cod, MA, was collected by Barnstable Animal Control in May 2021 and brought to the Cape Wildlife Center, where it was euthanized. Massachusetts Wildlife Services obtained the sample 2 weeks later and tested it using Direct Rapid Immunohistochemical testing (dRIT) as a part of routine enhanced surveillance (Rupprecht et al., 2014). The sample was confirmed positive by the CDC. Genomic DNA was extracted from brain or ear tissues using the DNeasy Blood & Tissue Kit (Qiagen) following the Tissues and Rodent Tails protocol on a QIAcube (Qiagen). The DNA concentration of extracts was measured using a Qubit 4 Fluorometer (Invitrogen Q33238).

### 2.2 SNP discovery

For initial SNP ascertainment, 380 raccoon samples collected in 2018 from the eastern U.S., ranging from Maine to Alabama, were submitted to Floragenex, Inc. for single‐end, restriction site‐associated DNA sequencing (RADseq) based on the SbfI enzyme and sequenced on an Illumina platform. Raw reads were demultiplexed using *process_radtags* in stacks2 (Catchen et al., 2013; Rochette et al., 2019). After demutiplexing, we mapped the reads to a draft raccoon genome (GCA_015708975.1; SAMN08536241; Tsuchiya et al., 2020) using the Burrows‐Wheeler aligner, bwa‐mem v0.7.17, with default settings (Li & Durbin, 2009). Single nucleotide polymorphisms (SNPs) were called with the *ref_map.pl* pipeline in stacks2, and the VCF (variant call format) file was generated using the *populations* command in STACKS2 with default parameters. The *populations* command was subsequently used to filter the VCF file for loci present in at least 50% of the population, a minor allele count of 3, and the ‐H setting to filter based on haplotypes. We used vcftools v0.1.17 (Danecek et al., 2011) to identify and remove individuals with greater than 50% missing data.

### 2.3 Microhaplotypes

We generated a BED (Browser Extensible Data) file from the VCF file using the SNP positions for assembling microhaplotypes, which we identified as multiple SNPs within a 150‐bp Illumina read. We used bedtools v2.30 (Quinlan & Hall, 2010) to isolate microhaplotype sequences by extending each SNP in the VCF file upstream and downstream 50 base pairs (bp) using the ‐*slop* command. We then merged “slop” loci by using the bedtools *merge* command set at 65 bp upstream and downstream. The merged loci were extracted from the draft raccoon genome FASTA file using the output from the *merge* command and the bedtools *getfasta* command. We mapped the raw RADseq data to the microhaplotype FASTA using bwa‐mem. To evaluate the quality of the microhaplotypes, we then recalled SNPs that were mapped to the microhaplotypes with STACKS2. We used vcftools for subsequent filtering of the microhaplotype VCF by repeating the filtering steps above, but we retained individuals with <25% missing data to get a broader diversity of microhaplotypes that may be better represented through genotyping with Rapture. We added an additional filtering step for the final VCF that included a maximum missing data per locus of 75%, a minimum mean depth per locus of 20×, and a minor allele frequency of 0.01. This final VCF file was used for microhaplotype genotyping using the microhaplot R package (Baetscher et al., 2018; https://github.com/ngthomas/microhaplot). We filtered microhaplotype loci in microhaplots based on 10× minimum depth and minimum allelic ratio of heterozygotes at 0.30. R scripts from https://github.com/PAMorin/Microhaplotype_processing were used to remove Ns from any microhaplotype genotype and replace it with missing data, retaining those with <20% Ns. The nucleotide sequences of the microhaplotype genotypes from the exported haplotable were manipulated using base R commands into a locus table, assigned numeric allele identifiers, and converted to genepop file format using the R packages adegenet v2.1.0 and hierfstat v‐0.5‐7 (Goudet, 2005; Jombart, 2008; Jombart & Ahmed, 2011). We evaluated population genetic summary statistics for each locus using adegenet and poppr v2.9.3 (Kamvar et al., 2014, 2015), which included heterozygosity, number of alleles, tests for linkage disequilibrium (index of association (*I*_A_) with 1000 permutations; (Agapow & Burt, 2001; Brown et al., 1980), and genotype accumulation curve to determine the minimum number of loci required to identify unique individuals. The remaining microhaplotypes that survived quality filtering were used for a hybrid capture design using MyBaits (Daicel Arbor Biosciences). Following quality filtering of the RADseq data, we used a VCF file containing 10,396 SNPs. After microhaplotype assembly and quality filtering, we ended up with 2475 microhaplotype loci that were in linkage equilibrium, for which multiple Rapture baits per microhaplotype were designed (mean number of baits per locus: 4 (range: 3–11); Tables S1 and S2).

### 2.4 RADcapture (Rapture)

We used 33 ng/μL of genomic DNA for Rapture genotyping and followed the protocol of Ali et al. (2015) with modifications. Following adapter ligation, we pooled 5 μL of each sample and purified the library with 1× Mag‐Bind TotalPure NGS Beads (Omega Bio‐Tek M1378). The pooled DNA was resuspended in 250 μL of low TE (ThermoFisher 12090015) and sheared in a M220 Focused‐Ultrasonicator (Covaris 500295). We used the default DNA_0300_bp_microTUBE_130ul_HolderXTU method and evaluated shearing efficiency with a fragment analyzer (QIAxcel; Qiagen). Additional shearing cycles were performed as necessary to achieve approximately 300 bp fragments. The resulting DNA was used in NEBNext Ultra DNA or NEBNext Ultra II Library Prep Kits for Illumina with modified purification of 0.75× Mag‐Bind TotalPure NGS Beads after PCR enrichment of adaptor‐ligated DNA. Targeted capture of the microhaplotype loci was completed using our custom baits and following the MyBaits Hybridization Capture for Targeted NGS standard protocol (Daicel Arbor Biosciences) using *T*_H_ and *T*_W_ of 62°. The resulting bead‐bound library was cleaned with 0.7× Mag‐Bind TotalPure NGS Beads and resuspended in 30 μL of low TE. The final library was quantified using the KAPA Library Quantification Kit (Roche KK4824), and 150‐bp reads were sequenced on a MiSeq (Illumina SY‐410‐1003) or NextSeq 550 (Illumina SY‐415‐1002), depending on the number of samples in the library. Microhaplotypes were assembled from the Rapture data following the approach outlined previously for the RADseq samples.

### 2.5 Statistical analyses

For the final microhaplotype dataset that contained 1160 raccoons (both RADseq and Rapture that included all samples from Maine to Alabama) and 1652 loci that met the filtering criteria in microhaplot, we implemented additional filtering: first, we removed loci missing more than 30% of genotypes; then we filtered individuals missing greater than 30% data; finally, we removed loci with minor allele frequency less than 1% using poppr. This left 819 polymorphic microhaplotype loci for 965 samples (the full dataset). We then removed 33 loci that had a sequencing depth greater than the upper 75% quantile of the mean depth, which resulted in a final 786 loci. The majority of the genotyped raccoons were used for a separate related study; however, to explore the qualities and diversity of the microhaplotype loci across all samples, we report some general statistics for the full dataset and then focus on the samples specific to this study.

**FIGURE 1:**
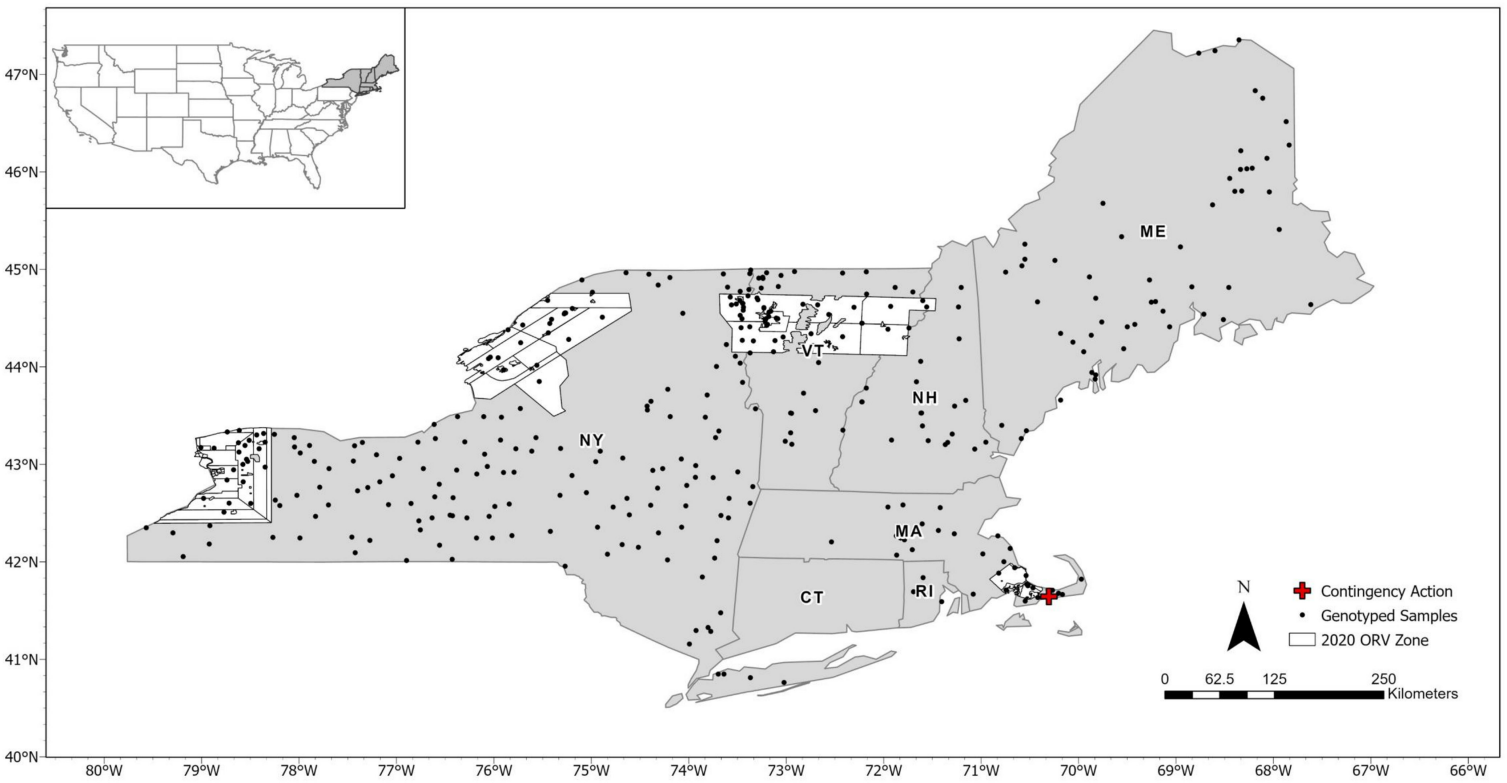
Map of sampling locations for raccoons (*Procyon lotor*) from the northeastern U.S. The locations in this map represent the NE‐NY dataset genotyped with RADseq and Rapture. The white areas are the 2020 oral rabies vaccination (ORV) zone. The red plus sign is the location of the rabid raccoon captured in the raccoon rabies variant‐free zone of Cape Cod, Massachusetts.

To evaluate hierarchical population structure in the northeastern U.S., we evaluated two datasets with samples from 2018 to 2021: one with 370 raccoons from six states (from here on the NE‐NY dataset): Maine (ME; *n* = 54), Massachusetts (MA; *n* = 36), New Hampshire (NH; *n* = 21), New York (NY; *n* = 201), Rhode Island (RI; *n* = 3), and Vermont (VT; *n* = 55; Figure 1); and a second with New York removed, leaving 169 individuals from five states (ME, MA, NH, RI, and VT; from here on the NE dataset). We used this approach because genetic clustering is often hierarchical, and focusing on a smaller geographic region may reveal additional population substructures not evident in broader sampling. The NE‐NY dataset consisted of 85 samples from the original RADseq dataset and 285 samples genotyped using Rapture, 277 genotyped on a Nextseq, and 8 genotyped on a Miseq (as part of a larger run that contained 47 samples). We estimated population genetic summary statistics for the full microhaplotype dataset as previously mentioned. We also estimated the probability of identity (*p*_ID_) and the probability of sibling identity (*p*_(ID)sib_), which are the probabilities that two randomly chosen individuals, or two siblings (*p*_(ID)sib_), have identical genotypes using the R package PopGenUtils with the command *pid_permute* and 1000 permutations (Tourvas, 2022).

To evaluate levels of population genetic structure within our datasets, we used the Bayesian clustering algorithm implemented in structure v2.3.4 (Pritchard et al., 2000), which forms groups of individuals based on Hardy–Weinberg and linkage equilibrium expectations. According to the software manual, estimating a unique allele frequency prior (*λ*) rather than running the default value of one can better suit datasets with low‐frequency minor alleles, which is the case for our microhaplotypes. We first estimated the value of *λ* with a run of *k* = 1 and 100,000 burn‐in and 200,000 MCMC chain lengths for the NE‐NY dataset. For the NE dataset, *λ* was estimated with a run of *k* = 1 with 50,000 burn‐in and 100,000 MCMC chain lengths. The resulting estimate of *λ* in the NE‐NY dataset was 0.4729, and for the NE dataset, it was 0.5113. With *λ* set at the estimated values, we searched for the optimal number of clusters (*K*) in the NE‐NY dataset by testing *K* from 1 to 15 with 20 replicates each, 50,000 burn‐in, and 100,000 MCMC chain length. For the NE dataset, we ran *K* = 1–15 with 20 replicates, 20,000 burn‐in steps, and a shorter MCMC chain length of 80,000 because of less variation in probability estimates in this dataset (see Figure S1 for convergence plots for both datasets). Given the uncertainty around estimating the true *K* in datasets with low genetic divergence, we used a combination of *K* estimators for each dataset that included Δ*K* (Evanno et al., 2005) and a parsimony estimator, *K*_PI_ (Wang, 2019), to identify a range of *K* estimates as a single estimator is often insufficient (Funk et al., 2020; Stankiewicz et al., 2022). Each of the *K* estimators was calculated using kfinder v1.0 and the web interface structureselector (Li & Liu, 2018; Wang, 2019). structureselector also initiates the clumpak algorithm, which evaluates the ancestry coefficient matrix (Q‐matrix) in the replicate runs, consolidates the replicates into a single matrix, and optimizes cluster label alignment for simplified comparison of ancestry across *K* (Kopelman et al., 2015). These matrices were then used to generate bar plots containing each individual's membership to inferred genetic clusters using the r package *structuRly* (Criscuolo & Angelini, 2020). An individual was included in a genetic cluster if >0.5 of its ancestry coefficient was assigned to a single cluster.

An additional nonparametric clustering analysis, discriminant analysis of principal components (DAPC) in adegenet (Jombart et al., 2010), was used to evaluate population structure. This method is free from population genetic assumptions about the underlying data, which are often violated in natural populations. DAPC uses information from multiple principal components (PC) to find the sample clustering that maximizes variation between groups while minimizing within‐group variation. We used the *a*‐score, which is the proportion of successful reassignment corrected for the number of retained PCs that assesses the power of assignment and limits over‐fitting, to determine the most optimal number of PCs (Jombart et al., 2010). The *a‐score* estimate of PCs can vary, so we calculated the a‐score 10 times and kept the minimum number of PCs identified as informative. We then used the *find.clusters* command to estimate the number of genetic clusters based on optimal PCs using *k*‐means, with the value of *k* chosen using the lowest Bayesian information criterion (BIC). The *k*‐means clustering was repeated 10 times to evaluate the variability in cluster assignment. Further, we used AMOVA, implemented in poppr, to quantify the amount of variance explained by hierarchical clustering of samples.

For each DAPC and structure genetic cluster in the NE‐NY and NE datasets, we estimated allelic richness in pegas with all defaults, except using the extrapolation method because of unbalanced cluster sample sizes (Foulley & Ollivier, 2006). Heterozygosity, private alleles, *F*_IS_, *F*_ST_ (*ϴ*; (Weir & Cockerham, 1984)) among clusters, and Hardy–Weinberg equilibrium (HWE) were estimated using adegenet, poppr, hierfstat, and diveRsity (Keenan et al., 2013). The HWE tests were first run on the NE‐NY dataset prior to clustering, and the separate genetic clusters for both datasets NE‐NY and NE datasets to evaluate if the number of loci that conform to HWE expectations increases as we divide the dataset among clusters, thus helping identify a Wahlund effect (Wahlund, 1928). All HWE tests were run for 1000 iterations, and *p*‐values were evaluated against a Bonferroni‐corrected *α* = 0.05. Using adegenet, we estimated genetic pairwise chord distance (Cavalli‐Sforza & Edwards, 1967) between individuals in the NE‐NY dataset and compared it to geographic Euclidian distance using a Mantel test to evaluate the significance of isolation‐by‐distance (IBD) (Mantel, 1967; Sokal, 1979).

## 3 RESULTS

### 3.1 Microhaplotypes

**FIGURE 2:**
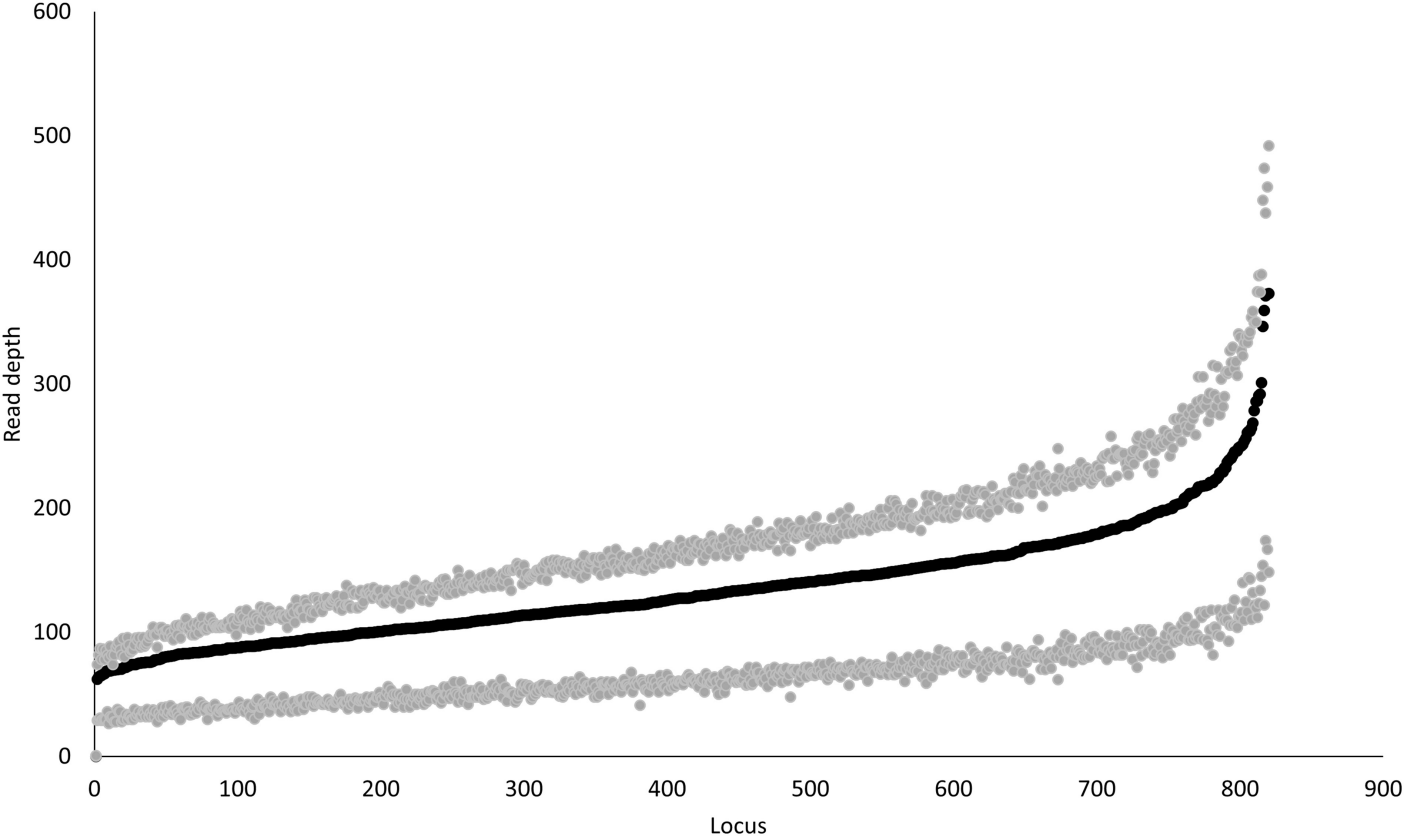
Distribution of read depth across 819 microhaplotypes genotyped in raccoons (*Procyon lotor*) using RADseq and Rapture. The black dots are the mean depth, and the gray dots are the 75% and 25% quartiles.

Tables S3–S5 present raw haplotypes, read depth, and mapping statistics to the original 2475 microhaplotype FASTA file for 817 samples sequenced by Rapture and 380 by RADseq (based on raw reads before quality filtering the full dataset). The mean number of reads per sample for the RADseq data was 3,634,010 (range: 19,571–22,227,479), and for the Rapture data, it was 433,031 (range: 654–3,143,232). The mean number of RADseq reads that mapped to microhaplotypes was 197,863 (range: 898–1,278,489), which was a mean proportion of 0.054 (range: 0.004–0.067) mapped reads. The Rapture dataset had a mean number of mapped reads of 97,135 (range: 175–531,699), which translates to a mean mapped proportion of 0.23 (range: 0.0025–0.43). Across all loci and samples, the mean number of SNPs per microhaplotype was 2.6 (range 1–8), resulting in a mean number of alleles per locus of 4 (range: 2–20; Figure S2). For the full dataset, which contained 786 microhaplotype loci and 965 raccoons, the mean missing data per locus was 10.3% and the mean depth per locus was 133× (Figure 2). Across the NE‐NY dataset (before clustering analyses), only 363 loci conformed to HWE. Significant departures from linkage equilibrium were not detected ($\bar{r_{d}}$ = 0.00551, *p* = 1). Using a genotype accumulation curve, we determined that 95% of the time, 137 loci can identify an individual animal, whereas 197 loci are required to identify all individual animals 99% of the time. Only 7 loci were required for a *p*_ID_ and 15 loci for *p*_(ID)sib_ less than 0.01 each.

### 3.2 New England/New York (NE‐NY dataset) structure and diversity

**FIGURE 3:**
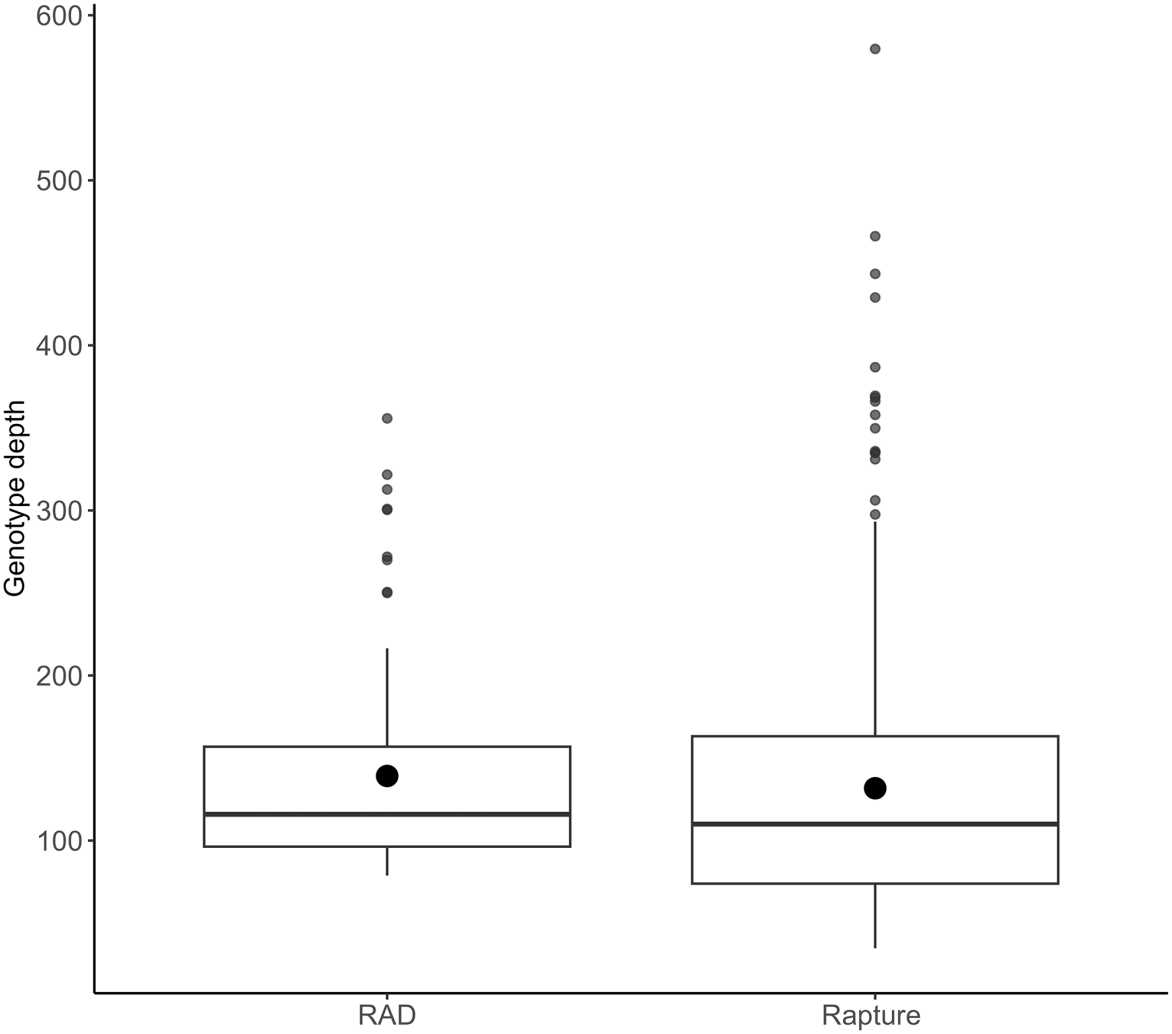
Boxplots of genotype depth for 819 microhaplotypes genotyped from raccoons (*Procyon lotor*). Each boxplot represents the depth of each sequencing method.

**FIGURE 4:**
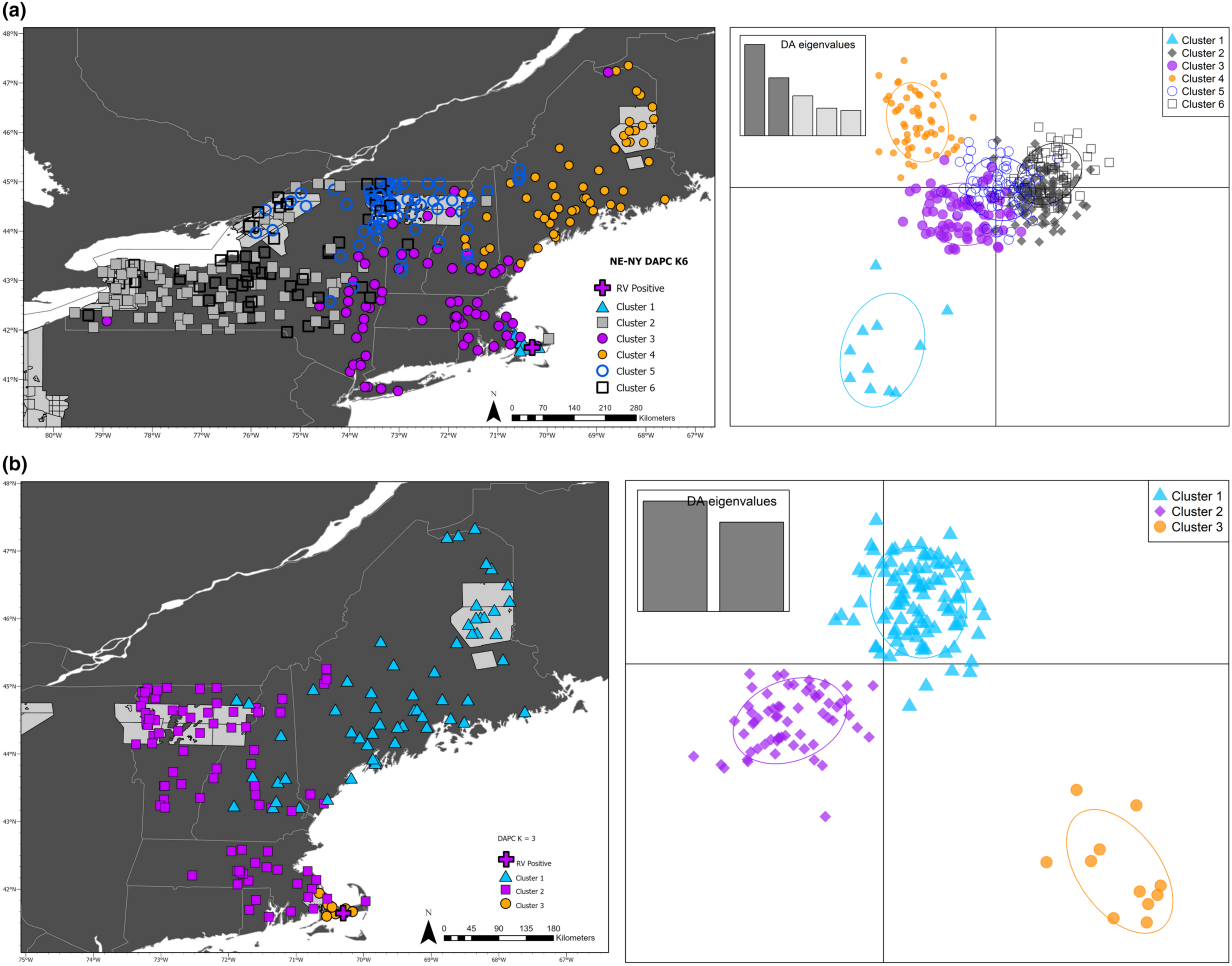
Results of genetic clustering using discriminant analysis of principal components (DAPC) for microhaplotypes genotyped in raccoons (*Procyon lotor*) from the northeastern U.S. Two datasets are represented, NE‐NY (a) and NE (b). For each dataset, the DAPC scatterplots are provided as well as a map with genetic clusters. The number of clusters (*K*) chosen for each figure was based on *k*‐means clustering. Colors represent the different genetic clusters identified with DAPC. The light gray areas on the map are the 2020 oral rabies vaccine (ORV) zone and the plus sign is the location of the rabid raccoon capture on Cape Cod, Massachusetts.

The mean, median, and quantiles of sequencing depth for 370 individuals in the NE‐NY dataset are presented in Figure 3 to demonstrate that the Rapture approach for genotyping raccoons generates equivalent depth as RADseq. Including the high‐depth loci, depth was similar for RADseq (mean: 139; range: 79–356) and Rapture (mean: 132; range: 34–580). Before analysis, we filtered the data to keep only the polymorphic loci, which resulted in 775 loci. The *a*‐score determined that 51 PCs (range: 51–53) contained the most information for DAPC and k‐means clustering. Six clusters were recovered from eight of 10 replicates using *k*‐means clustering, with the other two runs identifying *K* = 7. The cluster biplot and geographic locations for *K* = 6 are presented in Figure 4a. The genetic clusters occur in a continuum across the sampling area, with two overlapping clusters in NY, then three other clusters that occur mostly in northern NY and VT, one primarily in ME with a few individuals in NH, and a cluster that encompasses MA, eastern NY, and southern NH and VT. The final cluster is mostly located on Cape Cod, MA, with a few individuals on the adjacent mainland. The cluster numbers for each dataset and clustering approach throughout the manuscript do not directly align; therefore, these clusters will be referred to as D‐NE‐NY 1 through 6. After DAPC clustering, the number of loci that conformed to HWE expectations was 332 (42%).

Genetic diversity statistics for each of the six clusters were similar except the Cape Cod cluster (D‐NE‐NY‐1) had lower genetic diversity (Table 1). The mean genetic divergence among the six clusters was *F*_ST_ = 0.035 (range: 0.011–0.078), with the highest between the clusters that are the most geographically distant (Table 2; Figure 4a). The low genetic divergence among clusters is also demonstrated in the AMOVA, where 79% of the genetic variation is within individuals and only 2% is among clusters (Table 3). There were 127 loci that had private alleles, and each cluster had private alleles, with the most in D‐NE‐NY‐2 (Table 1; Figure 4a – gray cluster). The Mantel test over genetic and geographic distance matrices was significant (*p* = 0.005), suggesting IBD plays a role in population structure within northeastern raccoons, which is also evident from looking at the map, where all genetic clusters have some degree of overlap. In this dataset, the rabid Cape Cod raccoon belonged to cluster 3, which encompasses samples from southeastern New England, including MA, and eastern NY.

**TABLE 1:** Genetic diversity statistics for populations of raccoons (*Procyon lotor*) from the northeastern United States estimated from microhaplotypes.

Cluster	*n*	*H*_e_	*H*_O_	*P*_A_ (127 loci)	*A*_R_	*F*_IS_
NE‐NY DAPC K6						
D‐NE‐NY‐1	11	0.25	0.16	6	2.51	0.35
D‐NE‐NY‐2	93	0.3	0.26	117	3.45	0.15
D‐NE‐NY‐3	71	0.3	0.27	31	3.34	0.12
D‐NE‐NY‐4	55	0.29	0.26	25	3.18	0.12
D‐NE‐NY‐5	80	0.3	0.23	13	3.31	0.22
D‐NE‐NY‐6	60	0.31	0.25	22	3.33	0.18

Cluster	*n*	*H*_e_	*H*_O_	*P*_A_ (167 loci)	*A*_R_	*F*_IS_
NE‐NY Structure K4						
S‐NE‐NY‐K4‐1	49	0.29	0.25	5	3.14	0.12
S‐NE‐NY‐K4‐2	47	0.3	0.18	5	3.19	0.40
S‐NE‐NY‐K4‐3	110	0.3	0.27	124	3.48	0.11
S‐NE‐NY‐K4‐4	116	0.3	0.25	27	3.38	0.17

Cluster	*n*	*H*_e_	*H*_O_	*P*_A_ (147 loci)	*A*_R_	*F*_IS_
NE‐NY Structure K7						
S‐NE‐NY‐K7‐1	40	0.3	0.24	5	3.13	0.21
S‐NE‐NY‐K7‐2	31	0.3	0.28	14	3.15	0.07
S‐NE‐NY‐K7‐3	49	0.29	0.26	7	3.15	0.11
S‐NE‐NY‐K7‐4	7	0.27	0.2	5	2.43	0.28
S‐NE‐NY‐K7‐5	92	0.3	0.26	97	3.45	0.13
S‐NE‐NY‐K7‐6	26	0.29	0.16	6	3.00	0.44
S‐NE‐NY‐K7‐7	24	0.29	0.19	11	2.97	0.31

Cluster	*n*	*H*_e_	*H*_O_	*P*_A_ (246 loci)	*A*_R_	*F*_IS_
NE DAPC K3						
D‐NE‐1	100	0.31	0.25	211	3.27	0.19
D‐NE‐2	59	0.3	0.26	34	3.11	0.12
D‐NE‐3	10	0.26	0.18	7	2.43	0.32

Cluster	*n*	*H*_e_	*H*_O_	*P*_A_ (245 loci)	*A*_R_	*F*_IS_
NE structure K3						
S‐NE‐K3‐1	11	0.26	0.17	5	2.47	0.35
S‐NE‐K3‐2	99	0.31	0.26	216	3.27	0.17
S‐NE‐K3‐3	54	0.3	0.26	28	3.07	0.14

Cluster	*n*	*H*_e_	*H*_O_	*P*_A_ (179 loci)	*A*_R_	*F*_IS_
NE structure K4						
S‐NE‐K4‐1	47	0.3	0.24	50	3.09	0.21
S‐NE‐K4‐2	46	0.3	0.26	18	3.04	0.12
S‐NE‐K4‐3	60	0.3	0.27	107	3.20	0.14
S‐NE‐K4‐4	11	0.26	0.17	8	2.48	0.35

*Note*: The statistics are sample size (*n*), observed heterozygosity (*H*_O_), private alleles (*P*_A_) with the number of loci with private alleles in parentheses, allelic richness (*A*_R_), and inbreeding coefficient (*F*_IS_). Each group of statistics comes from a different data set and clustering approach (DAPC vs. STRUCTURE). The values are means except *P*_A_ which is the sum.

**TABLE 2:** Genetic divergence estimates based on the *F*_ST_ estimator *ϴ* for two raccoon microhaplotype datasets from the northeastern United States.

NE‐NY DAPC K6	D‐NE‐NY‐1	D‐NE‐NY‐2	D‐NE‐NY‐3	D‐NE‐NY‐4	D‐NE‐NY‐5	NE DAPC K3	D‐NE‐1	D‐NE‐2
D‐NE‐NY‐1						D‐NE‐1		
D‐NE‐NY‐2	0.073					D‐NE‐2	0.014	
D‐NE‐NY‐3	0.055	0.015				D‐NE‐3	0.056	0.071
D‐NE‐NY‐4	0.069	0.029	0.016					
D‐NE‐NY‐5	0.065	0.015	0.011	0.020				
D‐NE‐NY‐6	0.078	0.015	0.021	0.028	0.020			

NE‐NY Structure K4	S‐NE‐NY4‐1	S‐NE‐NY4‐2	S‐NE‐NY4‐3	NE Structure K3	S‐NE3‐1	S‐NE3‐2
S‐NE‐NY4‐1				S‐NE3‐1		
S‐NE‐NY4‐2	0.031			S‐NE3‐2	0.056	
S‐NE‐NY4‐3	0.029	0.017		S‐NE3‐3	0.069	0.014
S‐NE‐NY4‐4	0.017	0.021	0.017			

NE‐NY Structure K7	S‐NE‐NY7‐1	S‐NE‐NY7‐2	S‐NE‐NY7‐3	S‐NE‐NY7‐4	S‐NE‐NY7‐5	S‐NE‐NY7‐6	NE Structure K4	S‐NE4‐1	S‐NE4‐2	S‐NE4‐3
S‐NE‐NY7‐1										
S‐NE‐NY7‐2	0.018						S‐NE4‐1			
S‐NE‐NY7‐3	0.026	0.024					S‐NE4‐2	0.024		
S‐NE‐NY7‐4	0.034	0.040	0.049				S‐NE4‐3	0.013	0.016	
S‐NE‐NY7‐5	0.024	0.014	0.031	0.036			S‐NE4‐4	0.068	0.073	0.049
S‐NE‐NY7‐6	0.027	0.022	0.038	0.023	0.024					
S‐NE‐NY7‐7	0.033	0.032	0.035	0.057	0.039	0.047				

*Note*: The clusters were defined by two approaches, DAPC and STRUCTURE. The identification of the clusters is defined in the results.

**TABLE 3:** Analysis of molecular variance (AMOVA) for raccoon genetic clusters in the northeastern United States.

	Sigma	% variation	*φ*
NE‐NY DAPC K56			
Variation between clusters	0.9	2.0	0.0
Variation between samples within clusters	8.5	18.9	0.2
Variation within samples	35.7	79.1	0.2
Total variation	45.1	100.0	
NE‐NY Structure K4			
Variation between clusters	0.9	2.1	0.0
Variation between samples within clusters	8.3	19.9	0.2
Variation within samples	32.3	78.0	0.2
Total variation	41.5	100.0	
NE‐NY Structure K7			
Variation between clusters	0.9	2.9	0.0
Variation between samples within clusters	6.5	20.5	0.2
Variation within samples	24.1	76.5	0.2
Total variation	31.5	100.0	
NE DAPC K3			
Variation between clusters	1.2	2.7	0.0
Variations between samples within cluster	8.2	18.8	0.2
Variation within samples	34.2	78.5	0.2
Total variation	43.5	100.0	
NE Structure K3			
Variation between cluster	1.5	3.1	0.0
Variation between samples within populations	8.9	18.7	0.2
Variation within samples	37.2	78.2	0.2
Total variation	47.6	100.0	
NE Structure K4			
Variation between cluster	1.2	2.8	0.0
Variation between samples within populations	8.3	18.8	0.2
Variation within samples	34.6	78.4	0.2
Total variation	44.1	100.0	

*Note*: The clusters were identified using DAPC and STRUCTURE from microhaplotypes.

**FIGURE 5:**
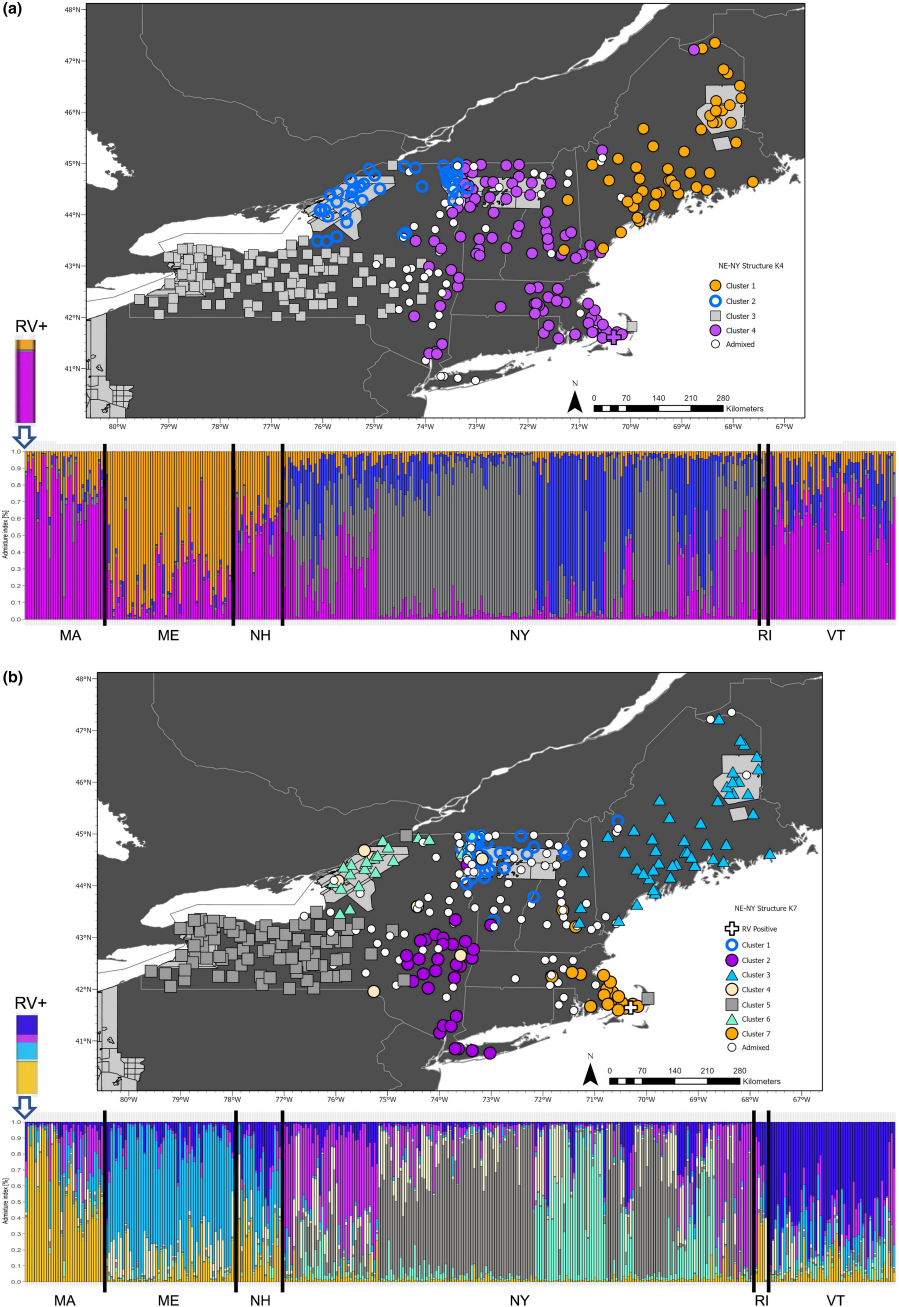
Results of genetic clustering using the Bayesian clustering algorithm in the software structure for microhaplotypes genotyped in raccoons (*Procyon lotor*) from the northeastern U.S. and representing the NE‐NY dataset. The ancestry coefficient bar plot is provided along with a map of genetic clusters. The number of clusters (*K*) chosen for each figure was based on the consensus of two *K* estimators (see Methods). Colors represent the different genetic clusters for *K* = 4 and *K* = 7. The light gray areas on the map are the 2020 oral rabies vaccine (ORV). RV+ represents the rabid raccoon.

**FIGURE 6:**
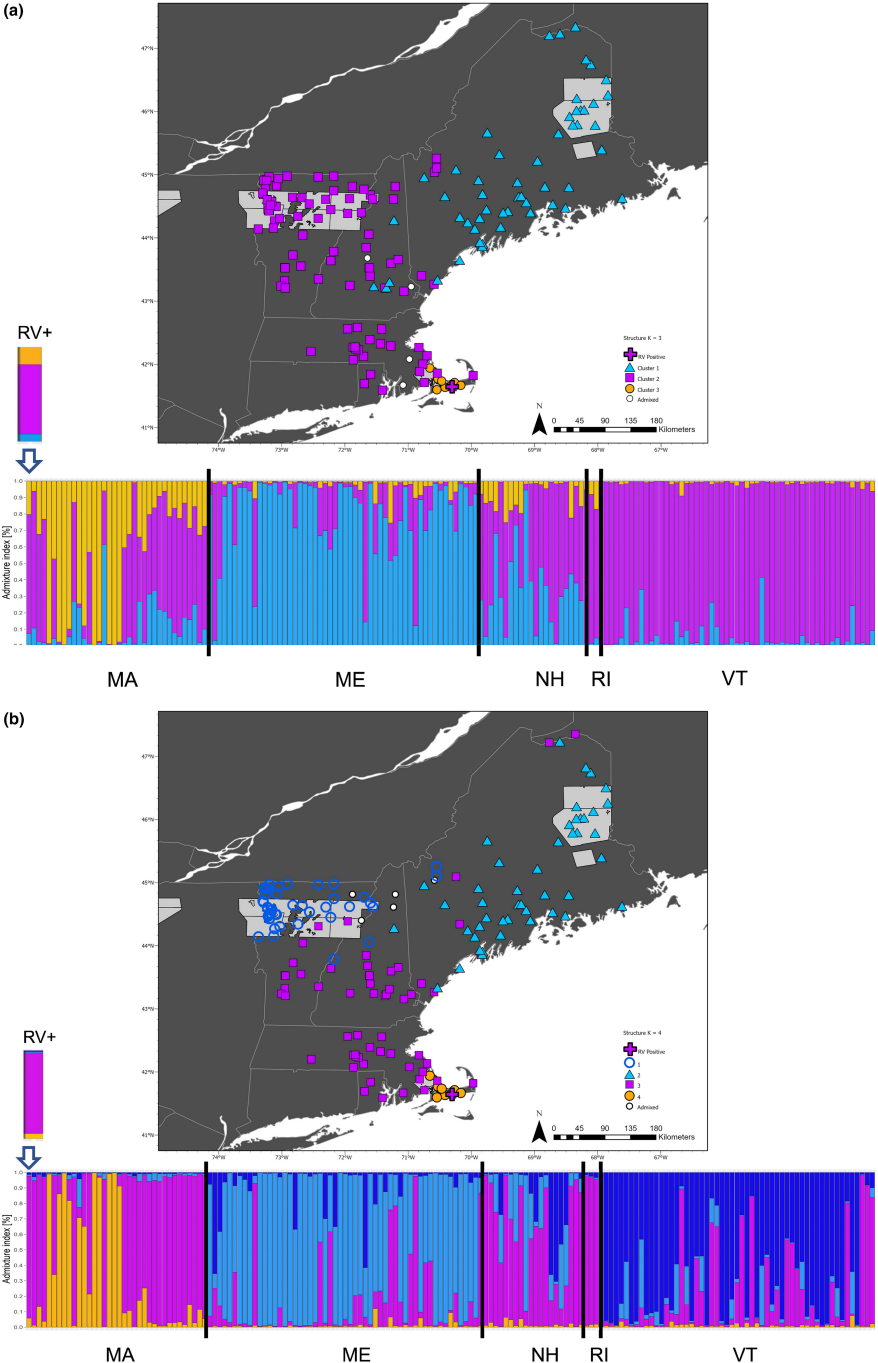
Results of genetic clustering using the Bayesian clustering algorithm in the software structure for microhaplotypes genotyped in raccoons (*Procyon lotor*) from the northeastern U.S. and representing the NE dataset. The ancestry coefficient bar plot is provided along with a map of genetic clusters. The number of clusters (*K*) chosen for each figure was based on the consensus of two *K* estimators (see Methods). Colors represent the different genetic clusters for the two competing numbers of clusters, *K* = 3 (a) and *K* = 4 (b). The light gray areas on the map are the 2020 oral rabies vaccine (ORV) zone and the plus sign is the location of the rabid raccoon capture on Cape Cod, Massachusetts.

The structure results for the NE‐NY dataset showed support for a range of *K*, depending on the estimator. Δ*K* showed the highest support for *K* = 2, followed by *K* = 4 and *K* = 7 (Figure S3). Given that Δ*K* has a tendency to show the strongest support for *K* = 2, despite the presence of more clusters, we will discuss results for other *K* values with the highest Δ*K* that also show support from multiple estimators (Janes et al., 2017). The parsimony *K* estimator, *K*_PI_, identified *K* = 7. At *K* = 4 (clusters S‐NE‐NY‐K4), most of the samples from ME form a cluster, the central New England states (MA, NH, VT) form a cluster, and there are two clusters in NY (Table 1; Figure 5a). The mean divergence among the *K* = 4 samples was *F*_ST_ = 0.022 (range: 0.017–0.032). There were 167 loci with private alleles. At this estimate of *K*, 346 loci (44%) conformed to HWE expectations. The AMOVA revealed that 78% of genetic variation was within individuals and 2.1% was among clusters. At *K* = 7 (clusters S‐NE‐NY‐K7), the predominant clusters were western NY (Cluster 5, *n* = 92), ME and eastern NH (Cluster 3, *n* = 49), eastern NY and southwestern VT (Cluster 2, *n* = 31), northern VT (Cluster 4; 1 = 40), northern NY along the Canadian border (Cluster 6; n = 26), and Cape Cod as well as the neighboring mainland (Cluster 7; *n* = 24; Figure 6b). The remaining samples were in cluster 4 (*n* = 7) which did not have a geographic association and was likely spurious or admixed with less than 0.50 membership coefficient to any cluster (*n* = 101).

The mean divergence among the *K* = 7 samples was *F*_ST_ = 0.032 (range: 0.014–0.057), with the highest values typically between Cape Cod (S‐NE‐NY‐K7‐7) and all other clusters. There were 147 loci with private alleles. At this estimate of *K*, 347 loci (44%) conformed to HWE expectations. The AMOVA revealed that 77% of genetic variation was within individuals and 2.9% was among clusters. The rabid raccoon sampled from Cape Cod (Figure 5b, white cross) clustered with the central New England cluster S‐NE‐NY‐K4‐4 at *K* = 4 and admixed with mainland raccoons at *K* = 7, not clustering with other Cape Cod racoon samples.

### 3.3 New England (NE dataset) structure and diversity

After removing the NY samples from the database for the NE dataset, we filtered to keep only polymorphic loci, which resulted in 758 loci and 169 samples for analyses. The DAPC analyses for the NE dataset were run with 41 informative PCs, and *k*‐means clustering divided the dataset into three clusters (Figure 4b). The clusters will be referred to as D‐NE 1 through 3. Like the broader analyses, all clusters have areas with overlap, but most geographic regions have a dominant genetic cluster. For example, ME is dominated by D‐NE‐1. In central New England, the dominant cluster is D‐NE‐2. The cluster D‐NE‐3 predominantly occurs on Cape Cod, with a few individuals on the nearby mainland. Like the NE‐NY dataset clusters, approximately 79% of the variation is within individuals (AMOVA; Table 3), and the genetic divergence is similar, with a mean *F*_ST_ of 0.047 (range: 0.014–0.071). The highest divergences were in the Cape Cod D‐NE‐4 cluster compared to the other clusters. Each cluster had private alleles, and in this scenario, 246 loci had private alleles. When focusing on the three NE DAPC clusters, 571 loci (73%) conformed to HWE expectations.

The structure analyses resulted in a variety of *K* supported by each of the estimators. The largest Δ*K* was at *K* = 3, followed by *K* = 4, but also showed strong support for *K* = 2 (Figure S4). *K*_PI_ estimated *K* = 4 as optimal. When evaluating the bar plots, *K* = 3 shows clusters associated with ME (S‐NE‐K3‐1; Figure 6a), central New England (S‐NE‐K3‐2), and predominantly on Cape Cod (S‐NE‐K3‐2), and *K* = 4 has nearly identical clusters except that northern VT becomes its own cluster (S‐NE‐K4‐1; Figure 6b). Geographically located between the clusters were admixed individuals. The structure analyses resulted in generally the same patterns as the DAPC clusters. For *K* = 3, 587 loci (75%) conformed to HWE, and at *K* = 4, 571 loci (73%) were in HWE. If we focus on *K* = 4, given the northern VT cluster was also identified in the larger NE‐NY dataset, we see that genetic diversity metrics, AMOVA results, and divergence of the clusters were similar to DAPC; mean *F*_ST_ = 0.040 (range: 0.013–0.073), with the highest between Cape Cod (S‐NE‐K4‐4) and all others (Tables 1, 2, 3). In both DAPC and structure analyses for the NE dataset, the rabid raccoon collected on Cape Cod clustered with the mainland clusters, S‐NE‐K4‐3 and D‐NE‐2, which were predominantly distributed in mainland MA, southern NH, and VT (Figures 4b and 6b).

## 4 DISCUSSION

We developed a rapid, cost‐effective HTS genomic approach to genotype raccoons for population genetic studies using benchtop Illumina platforms. We were able to quantify broad and localized patterns of raccoon population genetic structure in the northeastern United States. Delineating broad geographic clusters can set the stage for understanding how raccoons have historically moved, and contemporarily move across the landscape in the northeastern U.S. These movement patterns can then be used to guide rabies management, such as ORV zone movement, emergency response to new detections, and incorporation into epizootiological models to estimate risk and guide surveillance.

As sequencing costs decrease and the ease of computation improves, HTS will have increasing value for wildlife management and conservation programs. The main advantage of HTS over previous population genetic techniques is that sampling larger portions of a species' genome can increase the statistical robustness and precision of estimated population genetic parameters, which allows for stronger inferences about a species’ evolution and ecology (Allendorf et al., 2010; Shafer et al., 2015). However, for many questions, whole genomes, or even the tens of thousands of loci genotyped by reduced representation methods (ddRAD and RADseq), are unnecessary. For example, Schmidt et al. (2020) determined that a few hundred loci from GTseq resulted in genetic diversity and clustering estimates that were concordant with estimates from over 8000 RADseq loci. The ability to rely on fewer loci and the invention of rapid, cost‐effective genotyping approaches such as GTseq and Rapture allow for the use of HTS in situations where hundreds to thousands of samples need to be processed cost‐effectively or where samples of suboptimal quality are available.

The tradeoff when using equivalent numbers of bialleleic SNPs versus multiallelic loci, such as microsatellites, is the low information content in each locus, which requires significantly larger numbers of loci for the same level of resolution (Haasl & Payseur, 2011; Sunde et al., 2020). Microhaplotypes help reduce the number of loci needed when using HTS data for population genetics studies as they have more information content per locus than biallelic SNPs for inferences of population differentiation, structure, and ancestry (Baetscher et al., 2018; Bootsma et al., 2021; Gattepaille & Jakobsson, 2012; Mckinney et al., 2017; Morin et al., 2009). They also provide the promise of higher resolution and less subjectivity than microsatellites. Clustered SNPs that can be genotyped as microhaplotypes tend to be common throughout the genome, and when generating HTS data (such as RADseq), the presence of multiple SNPS on a single short read in collected datasets allows for genotyping panel development without additional specialized lab procedures (Kidd et al., 2013, 2014; Mckinney et al., 2017). In this study, we developed a Rapture protocol for rapid genotyping of raccoon microhaplotypes to help guide wildlife disease management.

### 4.1 Population structure

Through our analyses, geographic clustering of individuals was evident, and isolation by distance was significant. While the spatial coverage of our sampling could be improved in some areas (e.g., Western Massachusetts), we were able to identify population clusters throughout the northeastern U.S. connected by zones of admixture. The population structure of raccoons was also hierarchical, with substructures detected within each local region. Our results have provided additional information about the population structure in this region, as previous studies using a dozen or fewer microsatellites found low levels of population structure in small geographic areas and little influence of landscape features, aside from one study that found sex‐biased dispersal and another that found that the Niagara River impeded raccoon gene flow (Côté et al., 2012; Cullingham et al., 2009; Johnson et al., 2009; Root et al., 2009).

The hierarchical structure was evident not only from the clustering algorithms but also in the decreasing HWE deviations as the larger genetic clusters were evaluated at finer geographic scales, a likely result of a Wahlund effect (Wahlund, 1928). Ultimately, we ended up with around 20% of loci deviating from HWE in the NE dataset. Considering that HWE deviations decreased with more localized analysis, it is possible that a finer scale population structure exists. Other explanations for the HWE deviations that cannot be dismissed based on this study are selection or genotyping errors. Conducting further studies to understand what drives these deviations can highlight aspects of the data that are highly informative (Waples, 2014). We decided to keep the loci that deviated from HWE in the dataset given the potential for further Wahlund effects and that recent evidence suggests that filtering loci for HWE can lead to a reduced ability to detect population structure (Dharmarajan et al., 2013; Pearman et al., 2022). We also found individual clusters with greater numbers of private alleles compared to other clusters. Each of these clusters was on the edge of the sampling distribution, with western New York in the NE‐NY dataset and Maine in the NE dataset. We eliminated error‐prone individuals as the source of the private alleles. One possible explanation could be that these alleles are abundant outside our sampling region and that these areas have gene flow along the western and northeastern edges. Further sampling from Canada and nearby states, such as Pennsylvania, is needed to determine if this is the case.

The broadest regional dataset, NE‐NY, contained two to seven genetic clusters. When we compared the six DAPC clusters and the number of structure clusters with the most consensus of support, *K* = 7, we find good geographic correspondence (Figures 4a and 5). The clusters in ME contained nearly identical membership between the two methods. Where they differ is that the DAPC identified (1) two overlapping clusters in western NY, (2) northern NY and VT were a single cluster, (3) Cape Cod clustered with mainland samples, and (4) central MA, VT, and NH clustered with eastern NY. The DAPC had very few individuals that it identified as admixed, while structure was better able to determine levels of admixture. Further, structure identified additional clusters on Cape Cod and two clusters in Northern NY and VT separated by Lake Champlain. In this case, Lake Champlain appears to be a major impediment to raccoon gene flow. The cluster in northwestern NY along the Canadian border (Figure 5b, Cluster 6) also appears to be isolated in the south from other clusters. These data suggest that the higher elevation of the Adirondack mountains may be a barrier to raccoon movement, and other studies have shown that increasing elevation is negatively associated with raccoon abundance across the eastern U.S. (Slate et al., 2020).

The NE dataset was similar to the NE‐NY dataset in that its structure identified more admixed individuals and additional clusters than DAPC (Figures 4b and 6). structure also identified a cluster not detected by DAPC, with members from northeastern VT and northern NH (Figure 6b). Interestingly, the oral rabies vaccine (ORV) distribution zone in this area is almost on the southern edge of this cluster. Given that the dispersal distance of raccoons is less than 20 km, often less than 5 km depending on the habitat (Puskas et al., 2010; Rosatte et al., 2007, 2010), and that the individuals in northern VT share a region of contact south of the ORV zone suggest that animals move into this area but not commonly beyond. Thus, this area could be a possible candidate for ORV zone movement further south, as any infected animals that move into this area are unlikely to spread beyond it. Shifting the zone of vaccinated animals into an admixed area could limit the northward progression of RRV while meeting the goals of increasing RRV‐free areas in northeastern U.S.

### 4.2 Rabid raccoon on Cape Cod

In the NE‐NY *K* = 4 dataset, the rabid raccoon clustered with S‐NE‐NY‐K4‐4, which is central New England, and in the *K* = 7 dataset, the rabid raccoon was classified as admixed between three clusters. This pattern of admixture was also detected in the individuals from mainland MA, and central VT and NH (Figure 5b). When we zoom into the NE dataset, the admixed individuals in NE‐NY form a unique cluster (Figure 4b, Cluster 2 purple squares; Figure 6, purple squares) and the rabid raccoon clusters with this group of samples. Overall, the data suggest that the rabid raccoon originated on the mainland was not locally infected on Cape Cod, and that the microhaplotype approach is useful for identifying the origin of single samples.

Long‐distance human‐mediated movement is the likely origin of the original introduction of RRV to the mid‐Atlantic U.S. and may contribute to outbreaks within this region and Canada (Childs et al., 2000; Nadin‐Davis et al., 2020; Nettles et al., 1979; Rupprecht & Smith, 1994; Trewby et al., 2017). The risk of long‐distance movement of rabid animals is the impetus for this study, as further introductions across ORV zones to RRV‐free areas can compromise management efforts and bilateral progress toward local elimination along shared borders (Davis et al., 2023). Contingency actions to address the emergence or spread of RRV beyond management areas are costly, as detection of rabid mesocarnivores infected with the raccoon variant requires intense surveillance and possibly expensive trap–vaccinate–release efforts and high‐density distribution of ORV in the area (e.g., Lobo et al., 2018). Understanding whether a rabid animal was translocated, in the context of enhanced surveillance that did not detect any additional cases, helped the NRMP develop an effective yet cost‐efficient response that resulted in a significantly reduced time frame for vaccination and surveillance with no additional rabies cases.

### 4.3 Rapture and microhaplotypes

Both GTseq and Rapture are methods that reduce the cost of genomic data collection. We chose to use Rapture, as opposed to GTseq, because we wanted to test the ability to recover 2475 identified microhaplotypes in a single reaction, which is greater than the approximately 300–500 loci that a single GTseq primer pool can effectively amplify (Campbell et al., 2015; Meek & Larson, 2019). Multiplexing primers for more than 300–500 loci can be challenging, and the upfront costs of these primer pools can be in the thousands of dollars, whereas the cost of hybrid capture probes is similar to primer sets for around 1000 loci and can genotype many thousand more loci. When using probes in conjunction with the Rapture protocol of Ali et al. (2015), we cost‐effectively genotyped up to 96 samples in a single rapture pool and combined 288 individuals on a NextSeq and as many as 48 on a MiSeq, with a mean depth per locus of 136× for around 800 loci and a mean missing data per locus of around 10%. Bootsma et al. (2020) found that a depth of 31× was sufficient for reducing genotyping error to negligible levels. We effectively increased the number of reads that mapped to our microhaplotype loci from around 5.4% with RADseq to 24% using Rapture. According to a personal communication with the MyBaits manufacturer, around 30% of on‐target reads is typical. We compared reads mapped to our target microhaplotypes to the total raw reads before quality filtering steps. In the literature, on‐target recovery is often reported to be higher as final mapped reads are compared to post‐quality filtered data, but when compared to raw reads, the percent recovery of on‐target is similar to our findings (e.g., Sard et al., 2020). One proven way to reduce the cost even further is to develop a smaller panel of informative loci and use a PCR amplicon‐based approach such as GTseq (Meek & Larson, 2019; Sylvester et al., 2018). We plan to explore the use of this genotyping panel further by conducting additional raccoon population genetics studies in different regions of North America and validating the approach on lower quality samples (i.e., FTA cards and non‐invasive samples; e.g., Parker et al. (2022)) with the goal of providing ecological data to wildlife disease managers to guide management decisions.

Microhaplotypes for wildlife management and conservation are not widely used, but their use has been increasing over the last 5 years. Fully automated, user‐friendly pipelines for microhaplotype assembly, genotyping, and conversion to standard file formats (similar to STACKS2) are yet to be developed. Haplotype‐based callers, such as Freebayes and Stacks2, do generate haplotype‐based VCF files, but consolidating the haplotypes into a multialleleic format that is read by common population genetics software packages takes extensive manipulation. The r package, microhaplot (https://cran.r‐project.org/web/packages/microhaplot/), has helped with converting VCF to locus tables and has a very useful tutorial. However, following microhaplot, the conversion to a format that is compatible with analysis packages still requires some r or other command‐line formatting (see https://github.com/PAMorin/Microhaplotype_processing). A streamlined, user‐friendly microhaplotype pipeline would be a boon for broader use of this marker type, especially for labs that lack the computational expertise and infrastructure. Further, commonly used population genetic analytical approaches that are designed to efficiently process hundreds to thousands of loci, such as admixture (Alexander et al., 2009), only work with biallelic SNPs. The now classic program structure is useful for microhaplotypes as it was originally designed for multiallelic loci (this study, but also see Morin et al., 2021). structure can be computationally burdensome when running on local machines as it cannot run on parallel threads, and the GUI does not function on 64‐bit computers. But the development of packages that utilize the command‐line version to run in parallel on multiple threads, such as easyparallel and parallelstructure (Besnier & Glover, 2013; Zhao et al., 2020), helps reduce computation time. Recently, fineradstructure (Malinsky et al., 2018) has been applied to microhaplotypes, so this can be a good option for identifying genetic clusters with large datasets of multiallelic loci (Bootsma et al., 2021).

### 4.4 Future studies

Biological archives (biobanks) are a rich source of temporal and spatial epidemiological data (De Paoli, 2005; Kao et al., 2014). For example, Chandler et al. (2021) were only able to document the extent of white‐tailed deer (*Odocoileus virginianus*) exposure to SARS‐CoV‐2 because of access to a long‐term serum archive. To date, the NRMP has collected over 4000 raccoon samples from 23 states for the purpose of conducting genomic studies that can provide both basic and applied research that benefits rabies management. The NRMP is continually adding samples to the tissue archive from new states and areas, including the southern provinces of Canada impacted by RRV. In multiple areas, the sampling is dense enough to use these novel Rapture‐derived microhaplotypes to investigate population genetic patterns and social structure at a finer geographic resolution. While challenges in detecting population structure in raccoons at finer geographic scales may reflect a biological reality, the addition of landscape genetic approaches that account for isolation by distance, or using isolation by resistance models and multiple regression on distance matrices, genetic measures of relatedness data can be leveraged to identify the most important habitat corridors for regional RRV perpetuation and spread and to adapt and refine management targeting raccoon populations across rural and developed landscapes (Rioux Paquette et al., 2014).

## FUNDING INFORMATION

Funding for this study was provided by the United States Department of Agriculture.

## CONFLICT OF INTEREST STATEMENT

The authors have no conflicts of interest to declare.

## Supporting information


Figure S1.
Figure S2.
Figure S3.
Figure S4.

Table S1.
Table S2.
Table S3.
Table S4.
Table S5.

## Data Availability

The raw sequence reads are archived in the NCBI Sequence Read Archive under BioProject accession number PRJNA950910.
