# Supplementary material for: Are rabid raccoons (Procyon lotor) ready for the rapture? Determining the geographic origin of rabies virus‐infected raccoons using RADcapture and microhaplotypes
Source: Evol Appl. 2023 Nov 20;16(12):1937–55. doi: 10.1111/eva.13613 (PMC10739080; doi:10.1111/eva.13613)

Figure S1 – Plots of the alpha and F statistics from STRUCTURE 2.3.4 for the NE-NY dataset (A and B) and the NE dataset (C and D). Included are 10 replicates from *k* = 4 for both datasets. This demonstrates the that the MCMC chains converged before burn-in and they did not switch modes

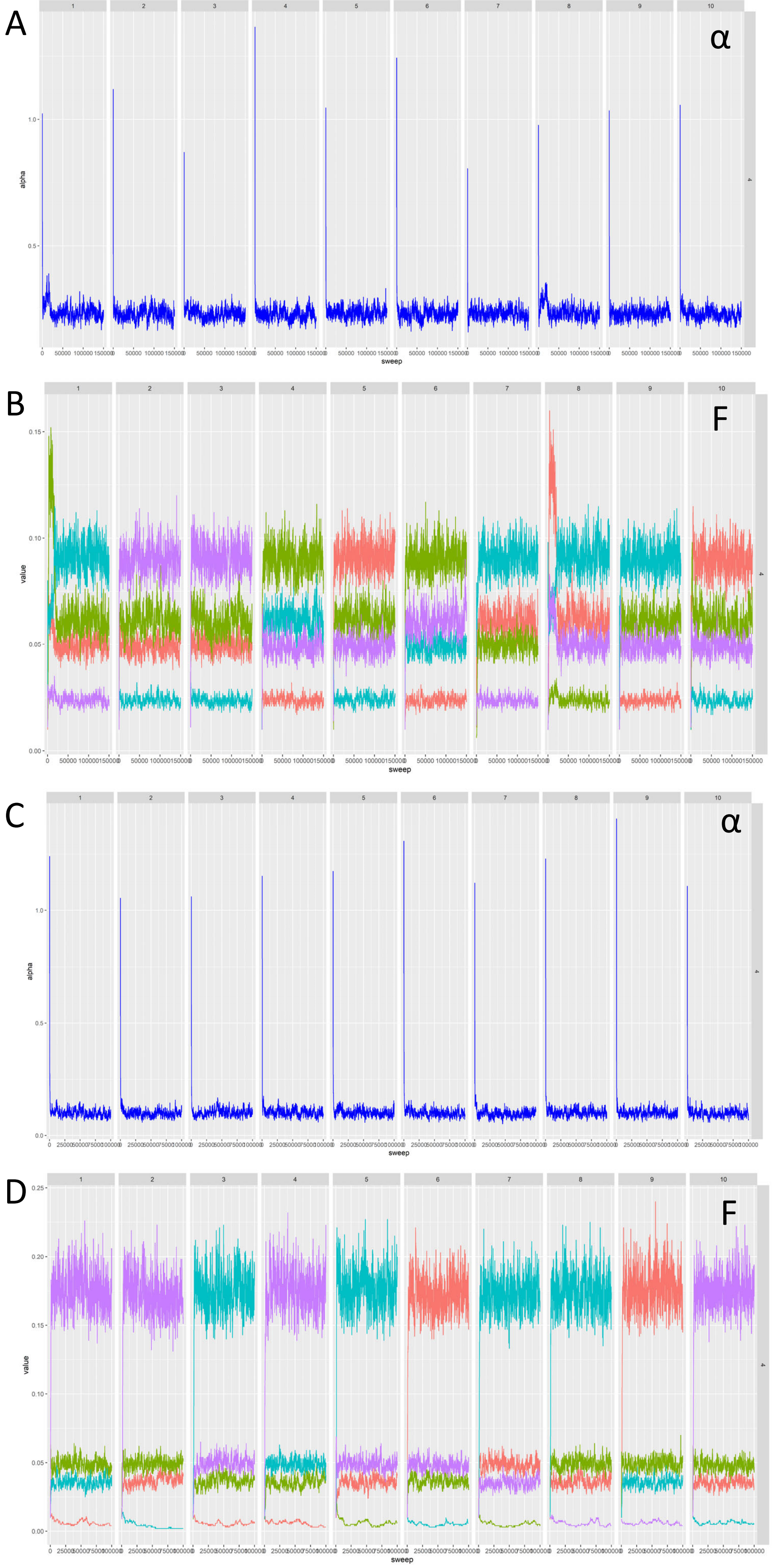

Figure S2 – A) Histogram of the number of SNPs per microhaplotype. B) Histogram of number of alleles per locus for 819 microhaplotypes genotyped in raccoons (*Procyon lotor*).

A

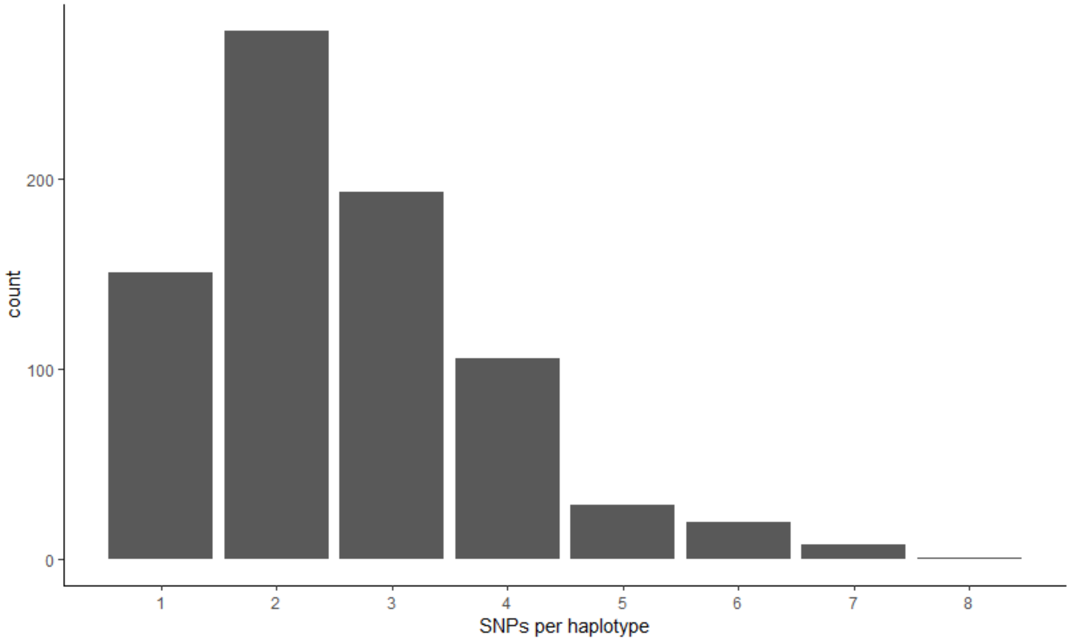

B

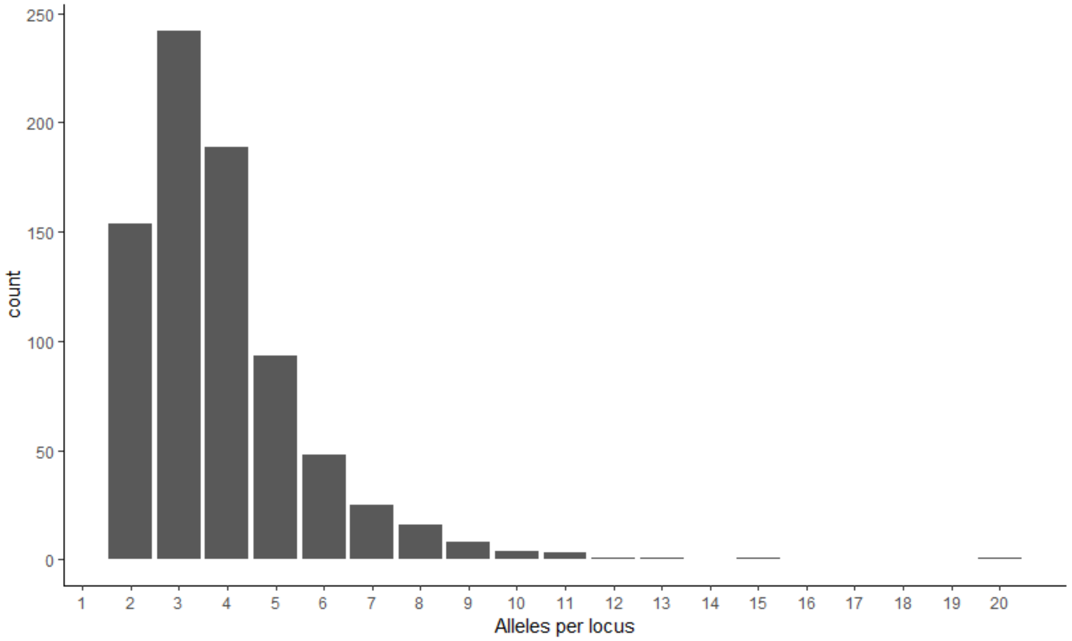

Fig. S3. NE-NY Structure Delta K

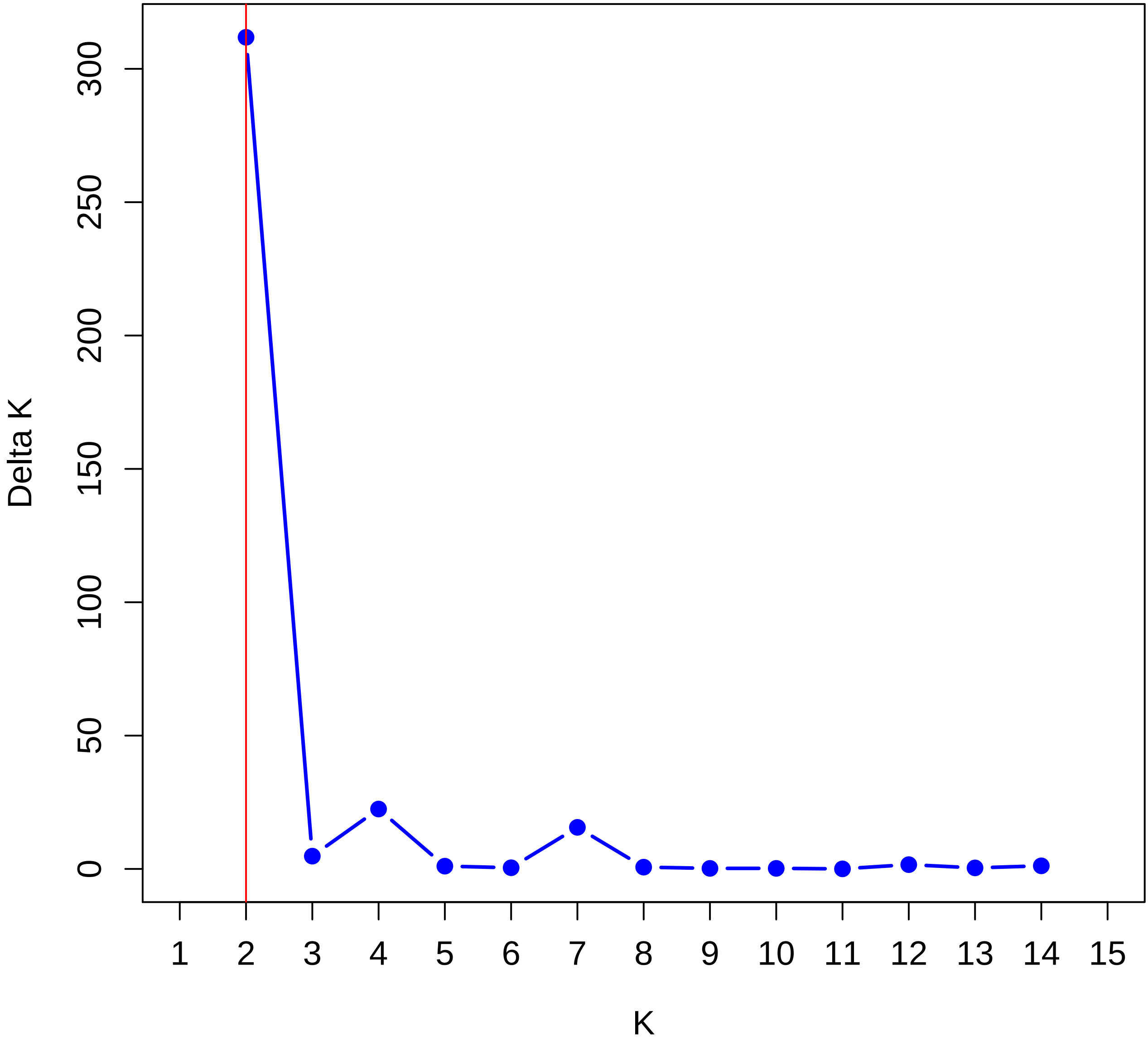

Fig. S4. NE Structure Delta K

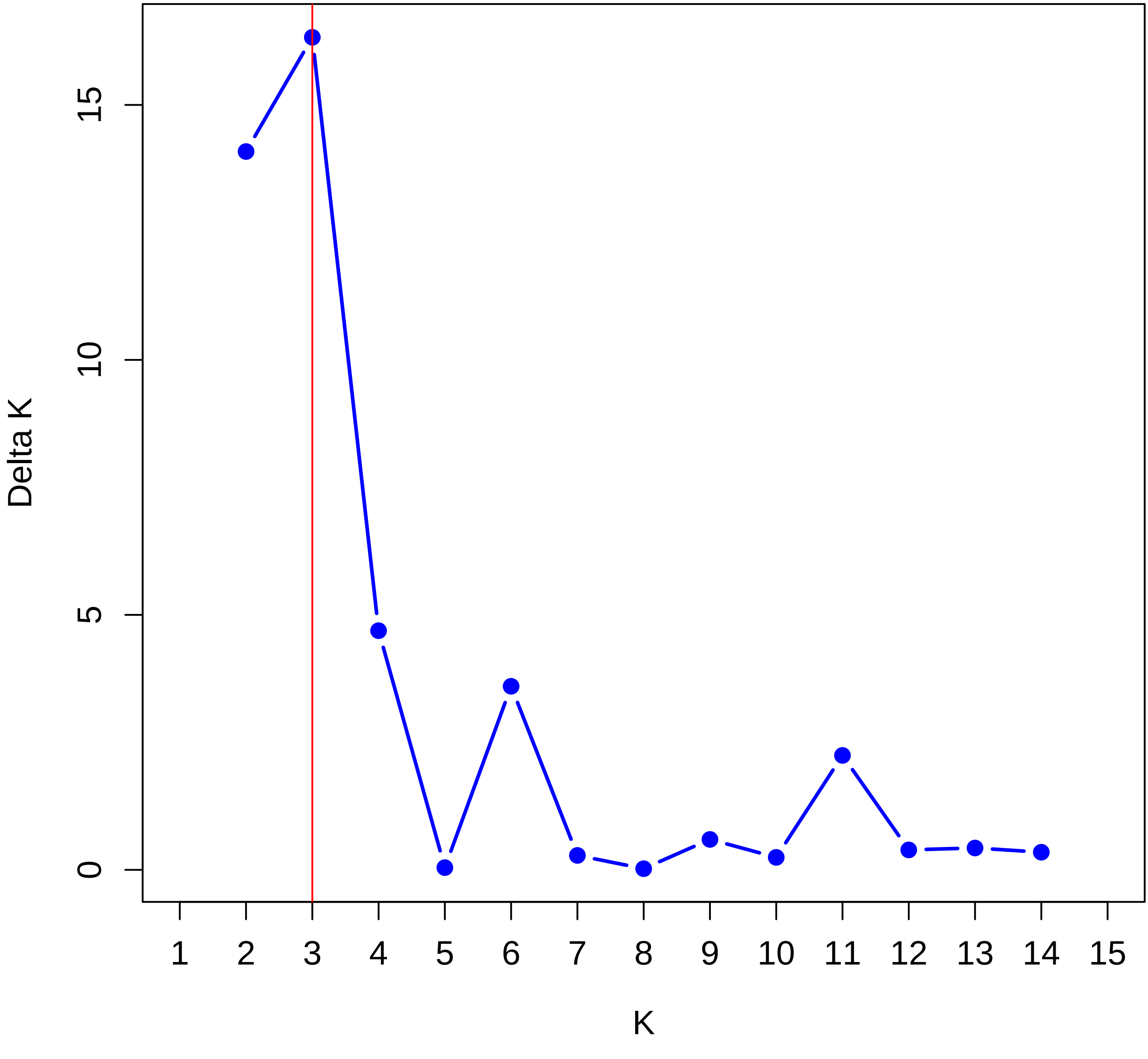

Supplement: Supplementary file 1 — Figure S1. Figure S2. Figure S3. Figure S4. [file EVA-16-1937-s001.pdf]
